# Ageing and osteoarthritis: a circadian rhythm connection

**DOI:** 10.1007/s10522-014-9522-3

**Published:** 2014-07-31

**Authors:** Nicole Gossan, Ray Boot-Handford, Qing-Jun Meng

**Affiliations:** 2 Wellcome Trust Centre for Cell Matrix Research, University of Manchester, Oxford Road, Manchester, M13 9PT UK; 1 Faculty of Life Sciences, University of Manchester, A.V. Hill Building, Oxford Road, Manchester, M13 9PT UK

**Keywords:** Osteoarthritis, Ageing, Circadian clock, Chondrocytes, Extracellular matrix

## Abstract

Osteoarthritis (OA) is the most common joint disease, affecting articular cartilage of the joints, with currently no cure. Age is a major risk factor for OA, but despite significant advances made in the OA research field, how ageing contributes to OA is still not well understood. In this review, we will focus on one particular aspect of chondrocyte biology, i.e., circadian rhythms. Disruptions to circadian clocks have been linked to various diseases. Our recent work demonstrates autonomous clocks in chondrocytes which regulate key pathways implicated in OA. The cartilage rhythm dampens with age and clock gene expression changes during the initiation stage of OA development in an experimental mouse OA model. Research into the molecular links between ageing, circadian clocks and OA may identify novel therapeutic routes for the prevention and management of OA, such as chronotherapy, or direct targeting of clock components/circadian rhythm.

Osteoarthritis (OA) is the most common joint disease, affecting over six million people and costing £5.7 billion annually in the UK alone (Arthritis Research UK, 2008). There is currently no cure, with treatments limited to painkillers and eventual joint replacement. OA primarily affects the articular cartilage lining the ends of long bones. Articular cartilage consists of abundant extracellular matrix (ECM), sparsely populated by chondrocytes. Isolated from the vasculature and lacking innervations, chondrocytes control cartilage tissue homeostasis by maintaining a fine balance between anabolic and catabolic activities. OA is characterised by a shift in chondrocyte homeostasis to a scenario in which catabolic activity outweighs anabolic activity, resulting in gradual degeneration of articular cartilage (van der Kraan 2012). The precise molecular mechanisms underlying disease initiation are unknown. OA is commonly referred to as a multifactorial disorder. Age is the single biggest risk factor for disease development; however OA is not inevitable in the elderly. Other risk factors (genetics, anatomical abnormality/mechanical injury and obesity) are thought to interact with age-related cartilage changes to initiate disease (Anderson and Loeser 2010). The aging process is thought to provide a ‘platform’ upon which other factors can more readily contribute to disease initiation, and as such a great deal of research has taken place into how chondrocytes and the cartilage ECM age.

## The circadian system

**Fig. 1:**
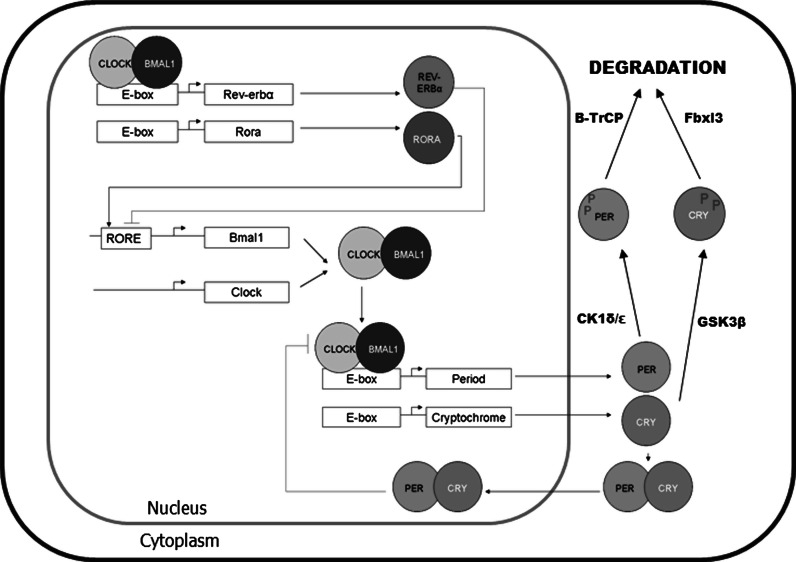
Molecular mechanism of the mammalian circadian clock. period (per1/2) and cryptochrome (cry1/2) genes are rhythmically transcribed by CLOCK:BMAL1 dimers. RORA and REV-ERBα rhythmically regulate BMAL1. Post-translational modification by casein kinases (CK1 enzymes) and GSK3β control the degradation of clock proteins. Altogether, this cycle occurs approximately every 24 h

The circadian (~24 h) clock in mammals is controlled by the central pacemaker in the suprachiasmatic nuclei (SCN) in the hypothalamus. The SCN integrates inputs from various external time-cues, predominantly light, in order to transmit temporal information to almost every cell and tissue in the body, and regulate genes controlling rhythmic tissue physiology (Takahashi et al. 2008). The molecular basis of circadian rhythm generation is increasingly well understood. In mammals circadian locomotor output cycles kaput/neuronal Per-Arnt-Single Minded domain-containing protein 2 (CLOCK/NPAS2) and brain and muscle Arnt like protein 1 (BMAL1) dimerise and activate transcription via E-box elements, including transcription of mammalian *Period* (Per 1/2) and *Cryptochrome* (Cry 1/2) genes. PER and CRY proteins accumulate and multimerise in the cytoplasm then move back to the nucleus to inhibit CLOCK/BMAL1 activity, thus inhibiting their own transcription (Fig. 1; Takahashi et al. 2008). This core transcriptional/translational feedback loop is modulated by other proteins including a stabilizing loop consisting of the orphan nuclear receptors retinoic-acid receptor related orphan receptor A (RORα) and REV-ERBα which bind to ROR response elements (ROREs) in the promoter of *Bmal1* to regulate its transcription. Post-translational modifications by kinases, such as the casein kinases (CK1δ/ε and CKII) and glycogen synthase kinase 3β (GSK3β), and phosphatases, such as protein phosphatase 2A (PP2A), regulate the activity and degradation of core clock proteins ensuring near 24 h cycles of activity (Fig. 1; Gallego and Virshup 2007; Meng et al. 2008a, b). Components of the molecular circadian clock control various target genes (clock controlled genes; CCGs) through response elements such as ROREs and E-boxes in their regulatory sequences.

**Fig. 2:**
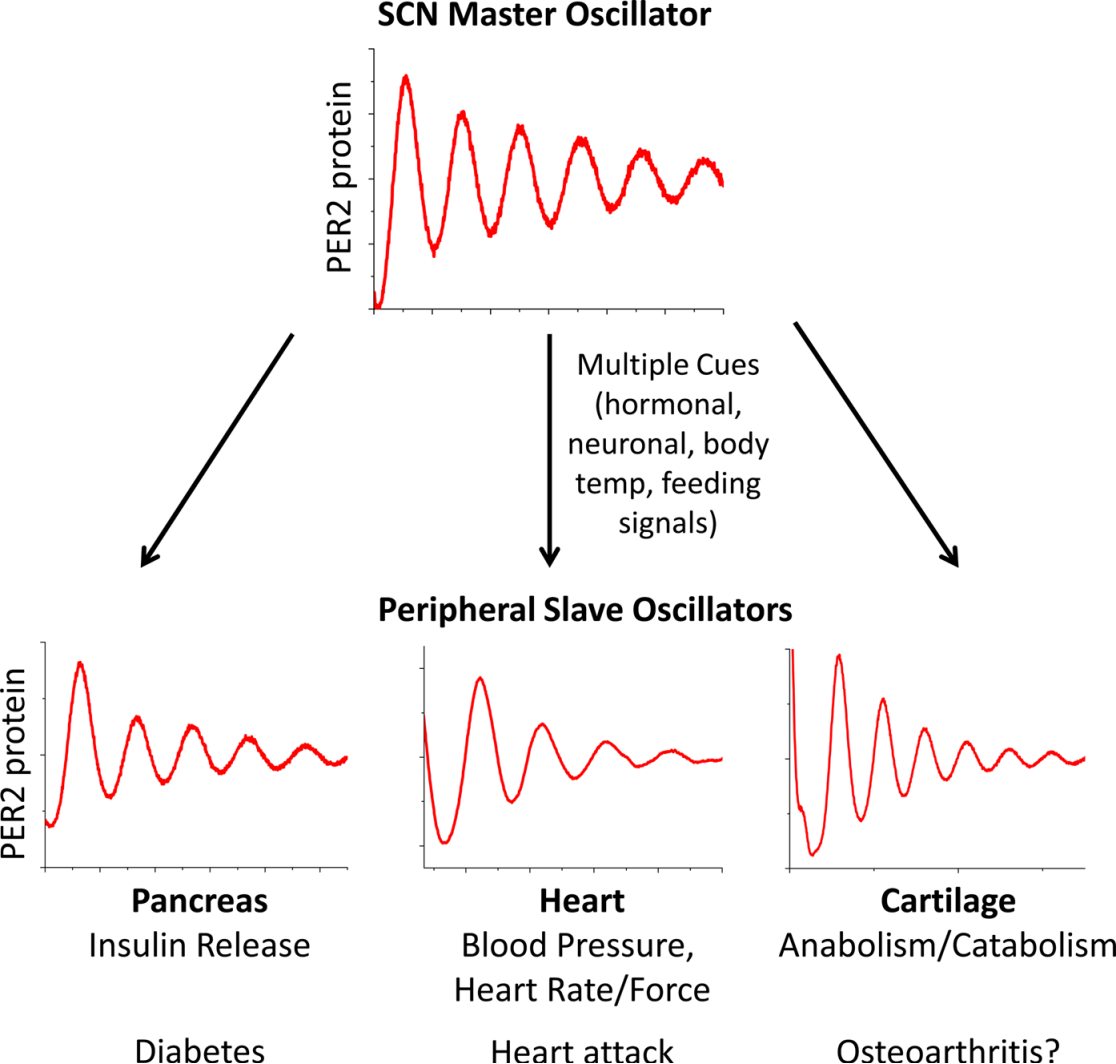
The master clock in the SCN controls rhythms in peripheral tissues to regulate physiology and pathology. Schematic representing rhythms in the SCN and peripheral tissues; multiple factors transmit timekeeping information from the SCN to set the phase of peripheral tissues; disruption of local clocks leads to changes in tissue specific outputs and causes a wide variety of health disorders

Autonomous clocks have been demonstrated in most peripheral tissues and cultured cells (Yoo et al. 2004; Balsalobre et al. 2000). The SCN is proposed as a master synchroniser of rhythms in peripheral tissues, ensuring their coordinated activity (Fig. 2). The precise nature of the SCN-controlled signal orchestrating peripheral clocks is an area of debate. Glucocorticoid signalling has been suggested as a candidate systemic entrainment factor, with rhythms in peripheral tissues of adrenalectomized rats demonstrating phase changes and desynchrony (Pezuk et al. 2012). There is also evidence that feeding signals entrain peripheral clocks, such as those in liver, colon and gut (Damiola et al. 2000; Sladek et al. 2007) and, recently, core body temperature rhythm has also been suggested as an important synchronising factor (Buhr et al. 2010), with exogenously applied temperature cycles approximating 24 h body temperature rhythms able to entrain peripheral tissues and cells (but not the SCN). It is likely that a number of signals act together to entrain peripheral clocks, perhaps with different signals being differentially salient to different tissues. Sujino et al. (2012) recently applied corticosterone injections and a restricted feeding schedule in antiphase on adrenalectomized rats. They showed that while the liver followed the phase entrainment of food cues, other tissues responded to corticosterone injection.

The circadian clock directs tissue physiology though control of tissue specific sets of CCGs. Microarray studies have revealed that up to 10 % of the transcriptome is expressed in a circadian manner, depending on the tissue and the stringency of the analysis (Panda et al. 2002; Hughes et al. 2009). Circadian genes are tissue specific (Yan et al. 2008); it has been postulated that circadian control of tissue specific master transcription factors, such as MyoD in muscle (Andrews et al. 2010) and KLF15 in heart (Jeyaraj et al. 2012), may be one explanation for this phenomena. The importance of circadian clocks in peripheral tissues has been highlighted in studies in which local clocks are conditionally ablated. Marcheva et al. (2010) ablated the clock specifically in pancreatic islets of mice. Despite the intact behavioural and SCN rhythm, these mice developed a diabetes mellitus-like disorder, in which insulin release and glucose tolerance was impaired. Similarly, tissue specific disruption of the circadian clock in the liver led to hypoglycemia and increased glucose clearance (Lamia et al. 2008), conditional clock disruption in adipocytes caused obesity (Paschos et al. 2012) and conditional disruption in the heart increased sensitivity to hypertrophy (Durgan et al. 2011). Further, mice with conditional *Bmal1* disruption in the retina responded abnormally to light (Storch et al. 2007) and those with conditionally arrhythmic macrophages lost the temporal gating of their response to endotoxin challenge (Gibbs et al. 2012). One caveat of these studies is that some phenotypes may be due to loss of one specific clock gene rather than due to arrhythmicity. For instance, although both *Bmal1* knockout and *ClockΔ19* mutant mice have disrupted CLOCK/BMAL1 activity and circadian rhythmicity, their phenotypes are strikingly different *Bmal1* knockouts demonstrate premature aging (Kondratov et al. 2006) whereas *ClockΔ19* mice are obese (Turek et al. 2005). This suggests possible clock-independent functions for at least some of the core clock transcription factors. Despite this, these studies underline the overall importance of circadian rhythms to tissue physiology.

## Circadian clocks in cartilage and chondrocytes

Until recently, evidence for a functional circadian clock in cartilage tissue capable of driving downstream CCGs has been largely circumstantial. Several lines of evidence indicate the circadian clock is involved in endochondral ossification, the process of bone formation from a cartilaginous template. Chondrocyte proliferation and growth plate height exhibit circadian variation (Stevenson et al. 1990), and measurement of calcium and phosphorus fractions from rat tibial growth plates indicated a higher rate of mineralisation during the dark-phase (Russell et al. 1984). A circadian rhythm in bone formation was confirmed using radiolabeled proline and galactose, which demonstrated that synthesis and secretion of ECM components by hypertrophic chondrocytes and osteoblasts exhibited a ~24 h rhythm (Igarashi et al. 2013). Osseointegration is the process by which bone implants become integrated with the surrounding tissue. A study investigating the effect of dietary vitamin D deficiency on osseointegration showed that clock gene expression was associated with expression of cartilage ECM markers (C*ol2a1, Col10a1* and link protein), all of which were increased at the implant site and associated with increased bone formation (Mengatto et al. 2011). Small interfering RNA knockdown of NPAS2 inhibited the induction of cartilage ECM genes produced by co-culturing MSCs with implant samples, while Sox9 expression was unaffected by the implant. This led the authors to speculate that during osseointegration the circadian clock contributes to ECM gene expression and subsequent bone formation. They suggest that CLOCK/NPAS2 may act directly on E-box elements in the promoters of *Col2a1*, *Col10a1*, and *Acan* (aggrecan). Their conclusion is supported by a study from Hinoi et al. (2006), which demonstrated the presence of circadian gene expression in femurs. The authors showed PTH injection could induce *Per1* expression, and identified a functional E-box in the first intron of *Col2a1.* Takarada et al. (2012) described shorter bone length and overall smaller body size both in *Bmal1 −/−* mice and in mice with a chondrocyte specific *Bmal1* deletion. The authors suggest that loss of circadian control of *Ihh* (Indian Hedgehog) in *Bmal1−/−* mice could underlie their reduced bone growth, and that a circadian clock in chondrocytes is necessary for appropriate bone formation. In in vitro studies, PER1 overexpression suppressed chondrocyte differentiation by suppressing a functional E-box in the *Ihh* promoter. Together, these studies suggest the importance of a cartilage clock during bone formation but they do not address the importance of this clock in later life, for example in the maintenance and repair of cartilage during aging and in disease.

Indeed, in resting cartilage (e.g., articular cartilage) the role of the circadian clock has been less well studied. Although it has long been known that suffers of both rheumatoid and OA experience time of day dependence in their symptoms (Bellamy et al. 1991, 2002), mainly indirect evidence has been provided for a role of the circadian clock in joint disease. An unbiased microarray study, which aimed to find mechanosensitive genes in an in vitro 3D sponge culture model of mechanical stimulation at a single time-point, identified the *Clock* gene in chondrocytes as being downregulated by mechanical stress (Kanbe et al. 2006). The authors also showed circadian expression of *Clock*, *Per1* and *Per2* in serum shocked chondrocytes, and that CLOCK protein expression is lower in late-grade OA cartilage than in earlier stages of the disease. Other studies have supported the concept that circadian clocks in joint tissues could be involved in disease progression. For instance, Haas and Straub (2012) showed that synovial tissue and cells from OA and RA patients did not demonstrate discernable circadian rhythms in clock protein or gene expression. Application of IL1β and TNFa increased clock gene expression in RA synovial fibroblasts, while they decreased expression in OA synovial cells. While this study suggests possible synovial clock disruption in OA and RA, it did not examine clock gene expression or the effect of cytokines on synovial fibroblasts from healthy controls, meaning that this work is far from conclusive. Further study by Kouri et al. (2013) compared RA and OA synovial tissue, using immunohistochemistry to show cytoplasmic localisation of BMAL1 in immune cells and fibroblasts in RA samples, while staining was nuclear in OA samples. Some clock genes were differentially expressed in OA and RA tissue, and in serum shocked fibroblasts from OA and RA patients. Again, the absence of healthy tissue samples for comparison has limited conclusions.

## Characterization of the cell-autonomous clocks in cartilage and chondrocytes

More recently, the PER2::luc clock reporter mouse model has been used in combination with microarray analysis, both by our group and by others, in order to unequivocally demonstrate an autonomous functional circadian clock in cartilage tissue explants and to identify its tissue specific targets. We used explants of permanent cartilage from PER2::luc mice to demonstrate the presence of an autonomous circadian clock in adult sternal cartilage, articular cartilage and in the femoral heads of newborn mice (Gossan et al. 2013). We also used lentivirus clock reporters to show that mouse and human chondrocytes contain autonomous clocks. Our findings of mouse cartilage rhythms were later confirmed by Okudo et al. (2013), in a study in which whole PER2::luc bones were cultured under bioluminescence imaging EM-CCD cameras. The strongest rhythmic bioluminescence signals originated from regions of both growth plate and articular cartilage, with limited expression of PER2::luc in the ossified bones. Expanding on this, we aimed to determine the synchronising factors which affect the phase of the cartilage clock (Gossan et al. 2013). We tested a synthetic glucocorticoid, dexamethasone, which is a known synchroniser of peripheral clocks, and found that it was able to rapidly and robustly reset the clock. Because cartilage is avascular, with nutrients diffusing to chondrocytes through the matrix, we speculated that body temperature signals might be a salient cue. To test this, we took two cartilage explants from PER2::luc mice, which were initially in phase. We incubated the tissues under opposite 12:12 h square wave temperature cycles, mimicking the typical high and low points of mouse body temperature. This is a crude model of body temperature oscillations in vivo, however when cultured under these conditions paired cultures from single mice were driven into antiphase. Phase changes were maintained when cultures were subsequently released into constant (37 °C) conditions. These data suggest that temperature is one way in which the SCN could communicate phase information with cartilage tissue. Melatonin, a circadian controlled hormone secreted by the pineal gland, has been suggested as a potential link between circadian rhythm and rheumatoid arthritis. This subject has been reviewed in detail recently (Yoshida et al. 2014). Another potential entrainment factor for the cartilage clock could be the daily loading/unloading associated with the rest/activity cycles, although experimental proof for this attempting hypothesis is currently lacking.

**Fig. 3:**
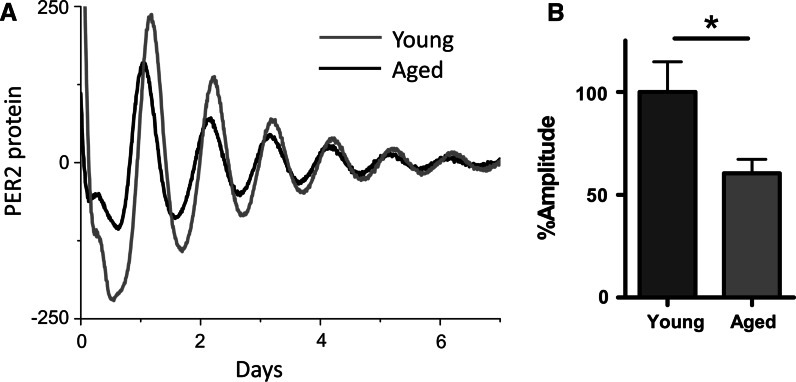
Age related decline in circadian amplitude in cartilage tissue explants. Adapted from Gossan et al. (2013): **a** representative trace of PER2::luc bioluminescence rhythms from xiphoid cartilage of young (2–4 months) and aged circadian reporter mice; **b** quantification of circadian amplitude showing a 40 % reduction in rhythm strength in aged (20–24 months) cartilage, mean ± SEM, *n* = 8 young, 11 aged, *t* test, *p* < 0.05

To demonstrate the function of the cartilage clock, as defined by its ability to drive the expression of a tissue specific set of downstream genes, we performed time-series whole genome microarray studies on cartilage tissue taken from mice kept in constant darkness over 2 days. The use of constant dark conditions eliminated effects of the light/dark cycle to reveal endogenously circadian genes which continued to oscillate in the absence of external cues. Six hundred and fifteen genes were identified as expressed in a circadian manner, comprising 3.9 % of total transcripts in cartilage tissue (Gossan et al. 2013; Fig. 3) including a number of genes with relevance to the pathogenesis of OA. Genes controlling apoptosis were rhythmic. Apoptosis increases in OA and is associated with proteoglycan and tissue loss (Lotz and Loeser 2012), while inhibiting apoptosis has been proposed as a therapeutic strategy (Kim and Blanco 2007). Specifically, the activated caspase three inhibitor *Xiap* has been identified as circadian. *Xiap* expression is antiapoptotic in OA chondrocytes (Bohm et al. 2010), with TNFα treatment leading to its downregulation (Yoshimura et al. 2006). Induction of *Xiap* expression is thought to at least partially underlie the protective effect of pulsed electromagnetic field therapy for post-menopausal OA (Li et al. 2011). Genes encoding ECM proteases were also identified as circadian, such as ADAMTS4 and MMP14 (Gossan et al. 2013). ADAMTS4 is one of the main proteases responsible for aggrecan degradation in articular cartilage (Verma and Dalal 2011). MMP14 is a membrane bound collagenase that is involved in regulating the pericellular ECM. MMP14 is also a key activator of other MMP proteases including MMP13 (Knauper et al. 1996), which is thought to be the main collagenase involved in OA. MMP14 knockout mice demonstrate articular cartilage destruction, accompanied by widespread skeletal pathologies (Holmbeck et al. 1999). Rhythmic control of anabolic genes and catabolic genes in chondrocytes could function to optimise cartilage repair/remodelling to optimum times of day; anabolic ECM genes such as fibrillins, laminins and netrin peaked in the early morning, along with catabolic genes controlling proteolysis. Restriction of ECM turnover to the early morning (in mice) may provide an efficient mechanism by which cartilage can recoup following bouts of nocturnal activity. More recently, Honda et al. (2013) used both permanent and growth plate cartilage from rats to perform time-series partial-genome arrays. Although a different statistical model was adopted to identify circadian regulated genes several were found to be common in rat and mouse (e.g., *Adamts4*, and others). Together, these recent studies demonstrate the presence of a functional circadian clock in cartilage tissue controlling pathways with importance in the cartilage tissue homeostasis and the development of OA.

## Ageing and cartilage rhythms

**Fig. 4:**
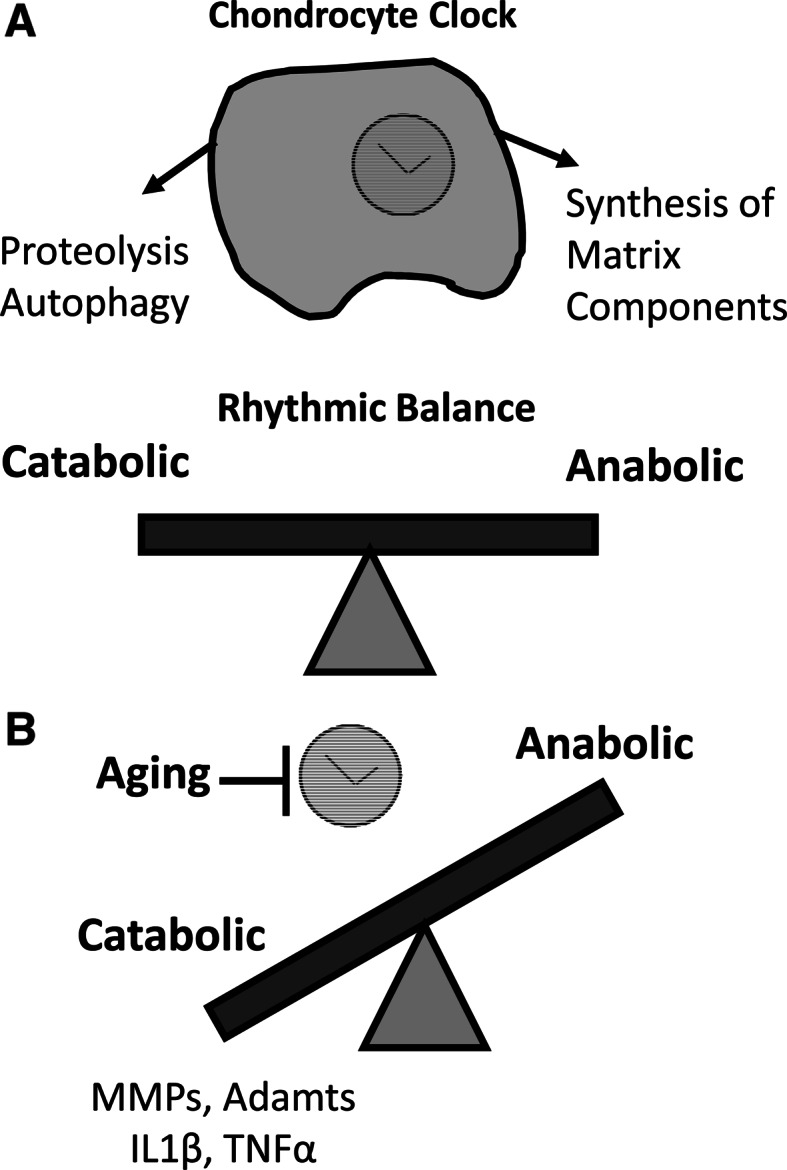
Age related imbalance of chondrocyte clock function. Simplified model showing the loss of homeostatic balance in chondrocytes during aging: **a** in normal physiology, a rhythmic balance of anabolic and catabolic activities ensures tissue remodelling and repair occurs optimally; **b** disruption to rhythmic balance during aging leads to excess catabolism and subsequent tissue damage

It is known that during aging, circadian rhythms deteriorate in a wide range of species, including humans. Through measurement of behavioural and physiological outputs such as sleep/wake cycles and body temperature or plasma hormone levels, it is generally agreed that age related circadian decline is manifest as a reduction in circadian amplitude (or strength) (for review see Brown et al. 2011). At the cell/tissue level, luciferase reporter studies suggest tissue specific effects of aging (Davidson et al. 2008; Sellix et al. 2012, and our own unpublished observations). Given the role of the chondrocyte clock in catabolic functions with potential relevance to OA, and the strong link between OA development and advanced age, we investigated how the circadian clock in cartilage changed during aging, which may contribute to increased OA susceptibility during aging. In cartilage tissue, we used PER2::luc explants to compare tissue oscillations from young and aged mice (Gossan et al. 2013). We found that the circadian amplitude of cartilage oscillations was significantly reduced in aged tissue. The decline in the circadian rhythm in cartilage could have a substantial impact on age-related OA susceptibility, due to loss of rhythmic expression of the 600+ previously identified cartilage CCGs controlling tissue homeostasis, matrix degradation and apoptosis (for a simplified model see Fig. 4). For instance, reduction in BMAL1 protein expression and attenuation of its circadian rhythm in aged articular cartilage could lead to increased cell senescence. Indeed, it has been reported aged chondrocytes demonstrate higher levels of stress-induced senescence (Loeser 2009). Senescent cells adopt a secretory phenotype, producing interleukins (IL6, IL1) and proteases (MMPs) which damage the matrix and contribute to OA (Anderson and Loeser 2010). Global *Bmal1−/−* mice demonstrate a premature aging phenotype, with widespread disruptions, including progressive arthropathy. One way in which this phenotype is thought to arise is through increased stress-induced cellular senescence (Kharpe et al. 2011); *Bmal1−/−* mice have increased ROS levels and demonstrate higher levels of cellular senescence in vivo, while *Bmal1−/−* cells are more susceptible to oxidative stress and DNA damage.

## SIRT1 as a potential link between ageing, clock disruption and OA

Of the pathways classically associated with aging, Sirt1 has received recent attention in the OA field because it is downregulated during aging and in OA in articular chondrocytes (Matsushita et al. 2013; Matsushita et al. 2013), and its disruption exacerbates experimental OA (Matsushita et al. 2013; Gabay et al. 2012; Gabay et al. 2013). Interestingly, an age related decline in Sirt1 has also been implicated as a cause for circadian disruption (Chang and Guarente 2013). It has been reported that SIRT1 interacts with the circadian system at multiple levels, through the direct regulation of *Bmal1* expression (Chang and Guarente 2013), and through PER2 and BMAL1 deacetylation (Asher et al. 2008; Nakahata et al. 2008). Further, the clock feeds back to regulate SIRT1 activity, through regulation of *Nampt* expression (Ramsey et al. 2009; Nakahata et al. 2009). NAMPT is the rate limiting enzyme controlling synthesis of the Sirt1 substrate NAD(+). Interestingly, we identified rhythmic gene expression of Nampt in cartilage tissue in our time series microarrays (Gossan et al. 2013). It is conceivable that during aging in cartilage tissue, changes in *Sirt1* expression and the circadian clock disruption could interact to increase OA susceptibility; SIRT1 disruption could lead to changes in the molecular clock and its downstream targets, or reciprocally changes to the circadian clock could disrupt SIRT1 activity by disrupting *Nampt* expression. Determining the nature of the relationship between these factors during aging would be an interesting area for future study.

In addition to the intrinsic decline of the chondrocyte clock, age-related changes in the systemic cues from the SCN may contribute to the dampened cartilage rhythm. It is thought that changes in the molecular rhythm in individual SCN cells (e.g., Sirt1 level, Chang and Guarente 2013), and changes in the synchrony between SCN neurones (Farajina et al. 2012), could reduce output amplitude during aging. For instance, the blunted cortisol rhythm during ageing could impact on the entrainment of the peripheral circadian clocks, including the chondrocyte clock (Kiessling et al. 2010). Indeed, Pagani et al. (2011) cultured skin fibroblasts from young and aged human volunteers. Using lentiviral delivery to express circadian luciferase reporters, they found no difference in the inherent clock properties of young and aged cells. However, application of serum from young and aged donors had significantly different effects on rhythm properties, suggesting that changes in a systemic factor underlie changes at the molecular level in skin cells. A reduction in systemic rhythmicity could also interact with OA risk factors in a variety of ways. Firstly, there is an intimate relationship between the circadian clock and metabolism. Disruption to the circadian clock, either genetically or environmentally, leads to increased propensity to obesity (Shi et al. 2013), which is a major risk factor for metabolic OA development (Zhuo et al. 2012). One of the ways in which obesity leads to metabolic OA is through increased systemic inflammation, another factor which is under circadian control (Scheiermann et al. 2013). Inflammation has also been shown to disrupt the expression of circadian clock genes in multiple tissues, including astrocytes, spleen and synovium fibroblasts (Cavadini et al. 2007; Duhart et al. 2013). It is conceivable that elevated systemic or local inflammation in the synovial joints, which is frequently observed in aged and OA joints (referred to as “inflammaging” of the joints, Berenbaum 2012), further contribute to the disruption of the cartilage clock. Finally, one of the most obvious outputs of the circadian clock is the daily cycle of rest and activity, affecting mechanical loading on joints. Misalignment of physical activity cycles with the optimum circadian phase, determined by local clocks in musculoskeletal tissues, could result in increased susceptibility to cartilage injury. Injury is one of the key initiating factors for OA development (Bhosale and Richardson 2008).

## Clinical implications of cartilage rhythms

The discovery of a functional circadian clock in articular cartilage, and that it declines during aging, opens up the possibility of targeting the clock to ameliorate age-related changes (and perhaps in OA-susceptibility). Chronotherapy, the concept of restricting the timing of drug treatments to maximise their efficacy and consequently reduce their toxicity, is a field which has grown rapidly in recent years with increasing experimental support (reviewed by Kaur et al. 2013). Currently, there is no disease modifying therapy available for OA. However, OA pain treatment has previously been shown to benefit from a chronotherapeutic dosing regimen (Levi et al. 1985). In future drug design, it is important to bear in mind the circadian nature of joint physiology and to determine whether or not drug targets are rhythmically expressed or active. In addition, targeting the circadian clock itself may be a useful approach to minimise or correct age related loss of cartilage function. Recently, compounds have been described which target molecular components of the clock such as the CRY proteins and REV-ERBs (Solt et al. 2012; Meng et al. 2008b; Hirota et al. 2012). Although these compounds are in the early stages of their development, they are likely to bring clinical benefits in the future. Given the well-established role of cortisol in entraining peripheral clocks, local delivery of synthetic glucocorticoids to the diseased joint may have potential therapeutic value to boost the intrinsic cartilage rhythm, in addition to their anti-inflammatory actions. In the meantime a systemic approach to strengthening circadian rhythms in the elderly may be useful, not only for improving cartilage function but for general health. Artificial lighting may not always be optimal for the endogenous circadian clock; exposure to bright sunlight during the day time and keeping bedrooms as dark as possible at night could be one way to strengthen SCN rhythms (Wright et al. 2013). Sticking to regular schedule of physical activity and eating meals at the same time each day could also be beneficial (Feillet et al. 2006; Hughes and Piggins 2012). The future identification of novel *zeitgebers* acting on the cartilage clock may be of interest here, for instance *Clock* has previously been identified as mechanosensitive (Kanbe et al. 2006; Simoni et al. 2014). If mechanical stimulation can re-entrain the cartilage clock, perhaps a regular schedule of gentle exercise would directly benefit the local cartilage clock in addition to having feedback effects on the central clock in the brain.

## Concluding remarks

Overall, the identification of a functional circadian clock in cartilage tissue (which dampens with age) has opened up an exciting new avenue for OA research. Future work should be directed towards understanding the major clock controlled pathways in cartilage in relation to OA. Moreover, it is important to demonstrate the functional significance of the cartilage clock using transgenic animals that lack chondrocyte clocks. Effects of clock-acting compounds on cartilage matrix homeostasis and disease progression of OA should be tested in vivo in experimental OA models. Epidemiological studies into sleep disorders and OA risks of the elderly population would further illuminate the functional links between human body clocks, ageing and OA.
